# Supplementary material for: Genomic landscape of virus-associated cancers
Source: Nat Commun. 2025 Jul 1;16:5887. doi: 10.1038/s41467-025-60836-9 (PMC12219571; doi:10.1038/s41467-025-60836-9)
Supplement: Supplementary file 1 — Supplementary Information [file 41467_2025_60836_MOESM1_ESM.pdf]

Genomic landscape of virus-associated cancers

Supplementary Figures

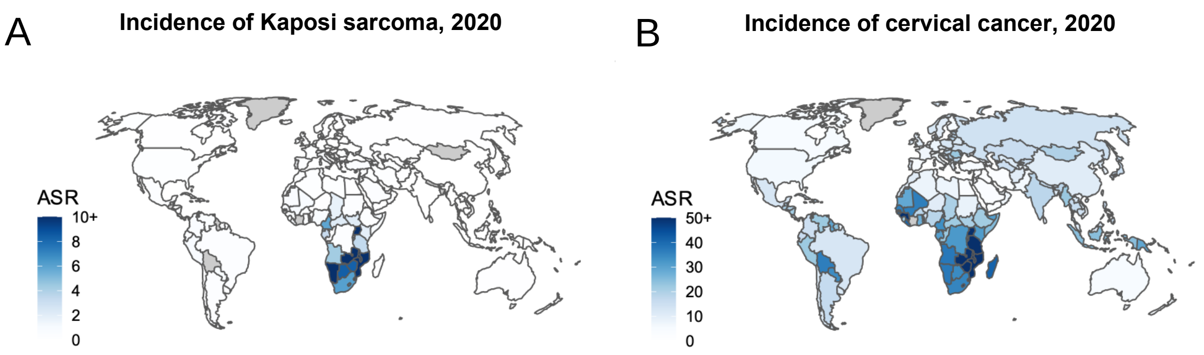

Fig. S1. Geographic distributions of Kaposi sarcoma and cervical cancer by country reported by GLOBOCAN 2020. A) Estimated age-standardized incidence rate (ASR) of Kaposi sarcoma by country. B) Estimated ASR of cervical cancer by country. Map data from Natural Earth (https://www.naturalearthdata.com/, public domain), produced by rnaturalearth R package[195].

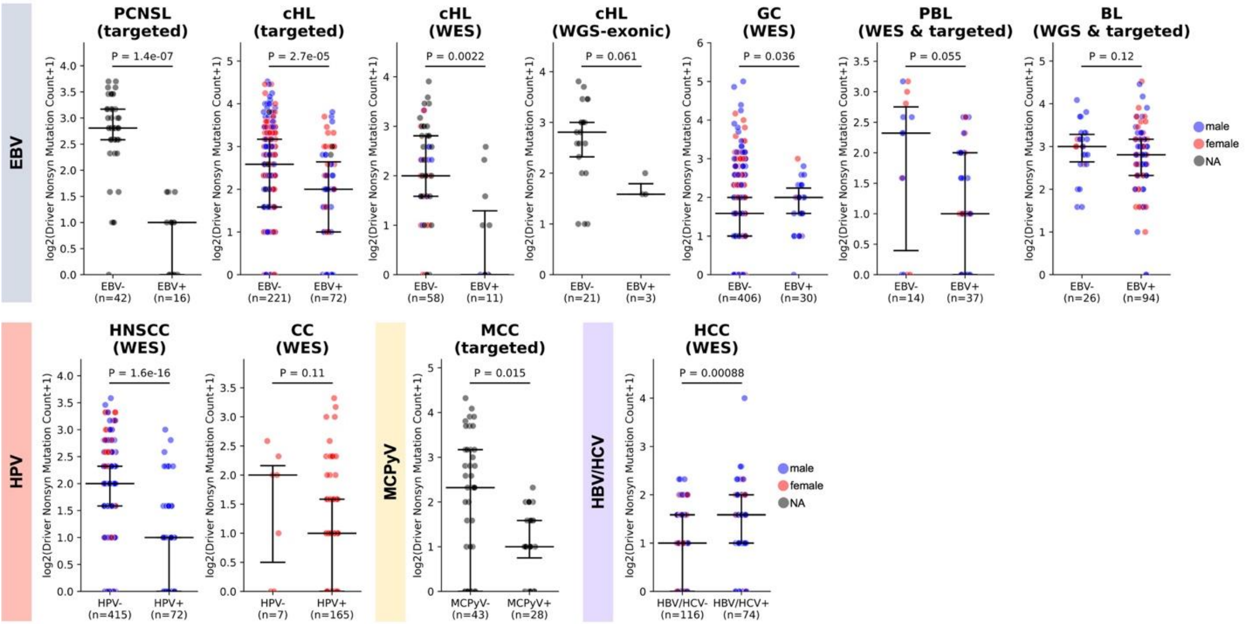

Fig. S2. Driver mutations in virus positive vs. negative tumors in nine cancers. P values from two-sided MWU test. Data are presented as median values with interquartile range (25th–75th percentile). GC, gastric cancer; HCC, hepatocellular carcinoma; cHL, classical hodgkin lymphoma; HNSCC, head and neck squamous cell carcinoma; MCC, Merkel cell carcinoma; PBL, plasmablastic lymphoma; PCNSL, primary central nervous system lymphoma; CC, cervical cancer; BL, Burkitt lymphoma. Source data are provided as a Source Data file.

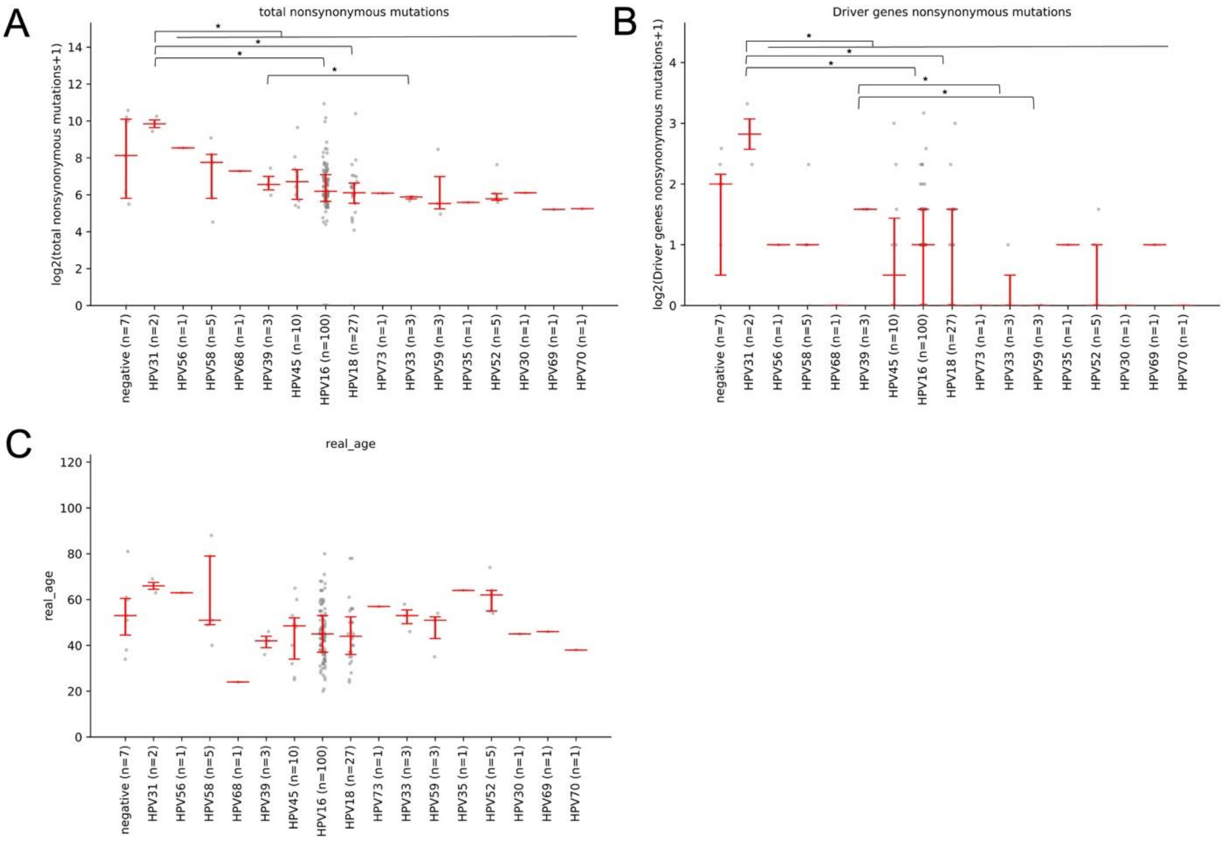

Fig. S3. Cervical cancer HPV strain differences in A) total nonsynonymous mutation load, B) driver gene mutation load, and C) age at disease onset. Refer to Table S6 for significant associations between age and HPV strains. Data are presented as median values with interquartile range (25th–75th percentile). * two-sided MWU test, $p < 0.05$. Source data are provided as a Source Data file.

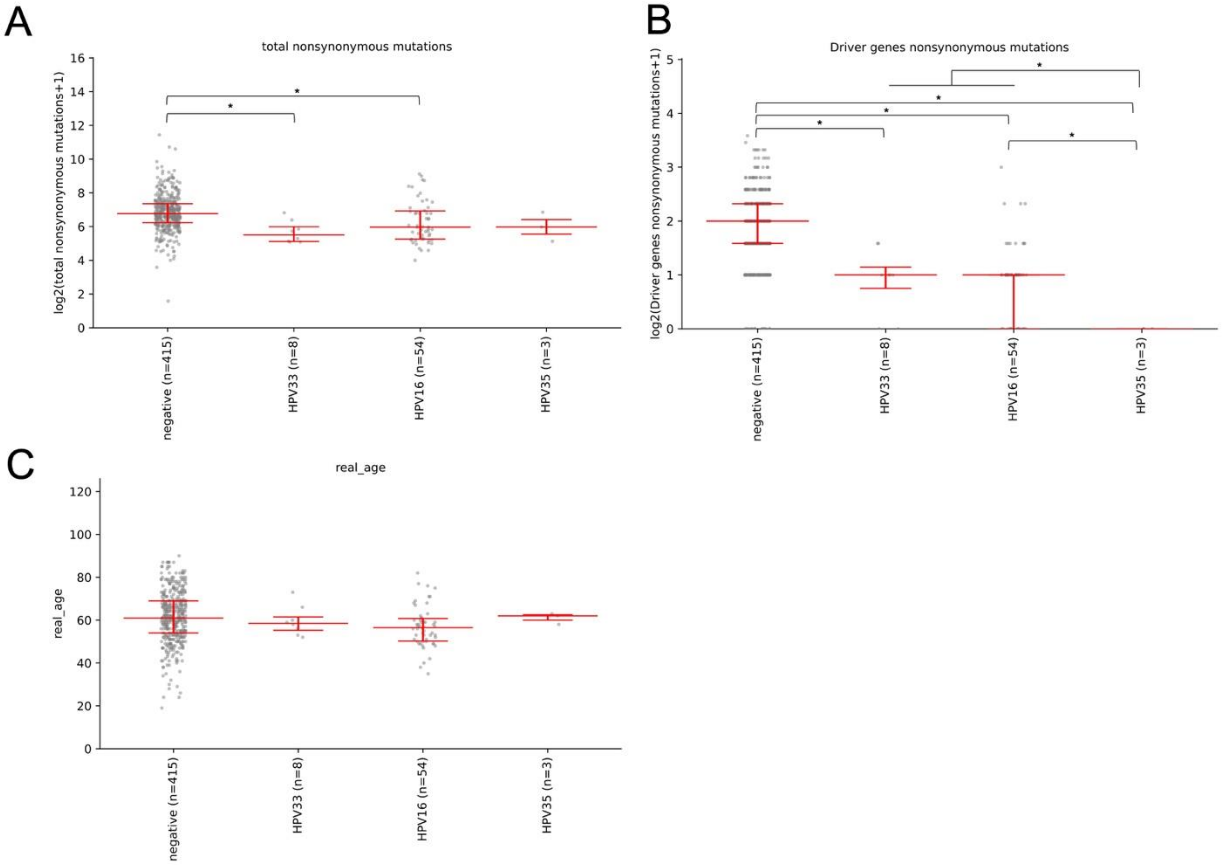

Fig. S4. HNSCC HPV strain differences in A) total nonsynonymous mutation load, B) driver gene mutation load, and C) age at disease onset. Data are presented as median values with interquartile range (25th–75th percentile). * two-sided MWU test, $p < 0.05$. Source data are provided as a Source Data file.

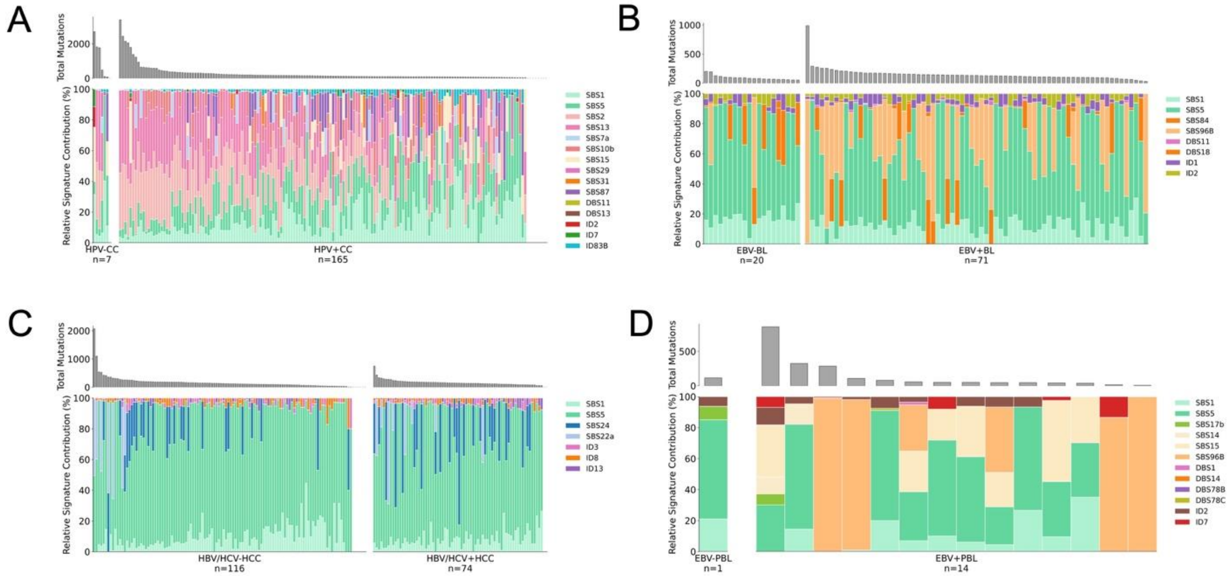

Fig. S5. Mutation signatures in virus-associated cancers. A) CC (n = 172), B) BL (n = 91; 68 EBV-positive eBL, 6 EBV-negative eBL, 3 EBV-positive sBL, 14 EBV-negative sBL), C) HCC (n =190), D) PBL (n = 23). HCC, hepatocellular carcinoma; PBL, plasmablastic lymphoma; CC, cervical cancer; BL, Burkitt lymphoma. Source data are provided as a Source Data file.

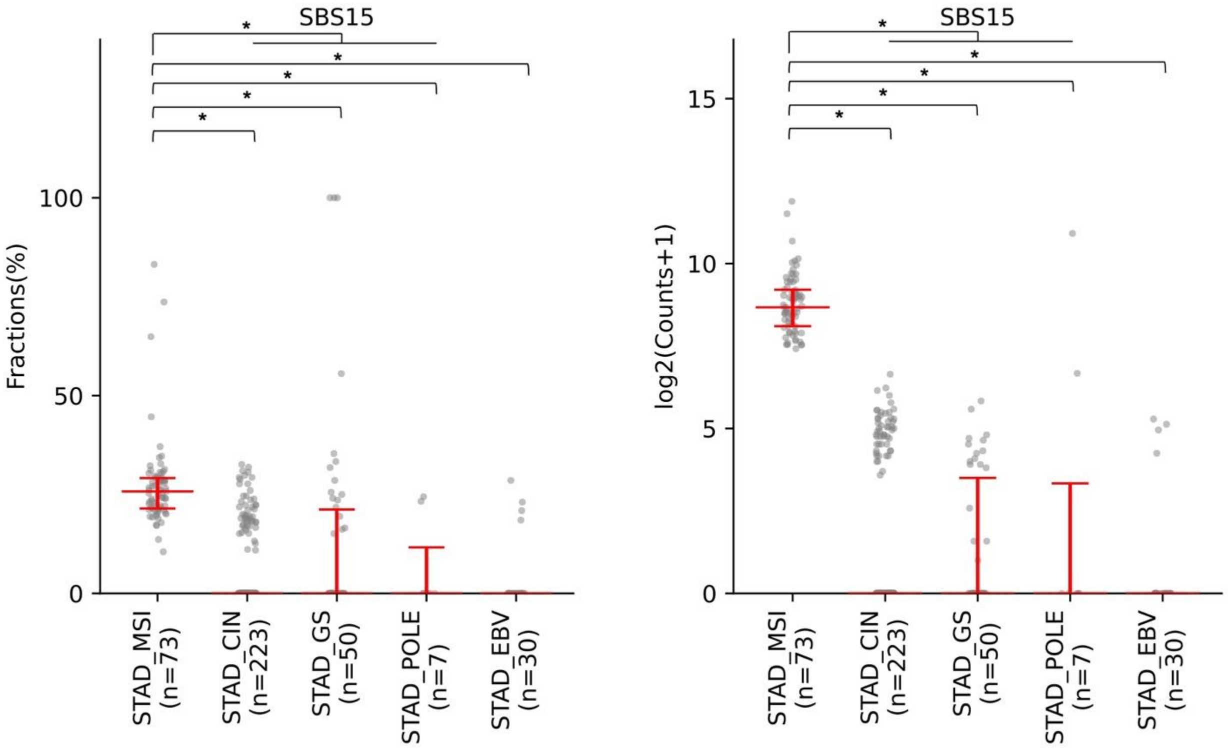

Fig. S6. Gastric cancer subtype differences in A) fractions (%) and B) log2(mutation counts+1) of SBS15/MMR-deficient signature. Data are presented as median values with interquartile range (25th–75th percentile). * two-sided MWU test, $p < 0.05$. Source data are provided as a Source Data file.

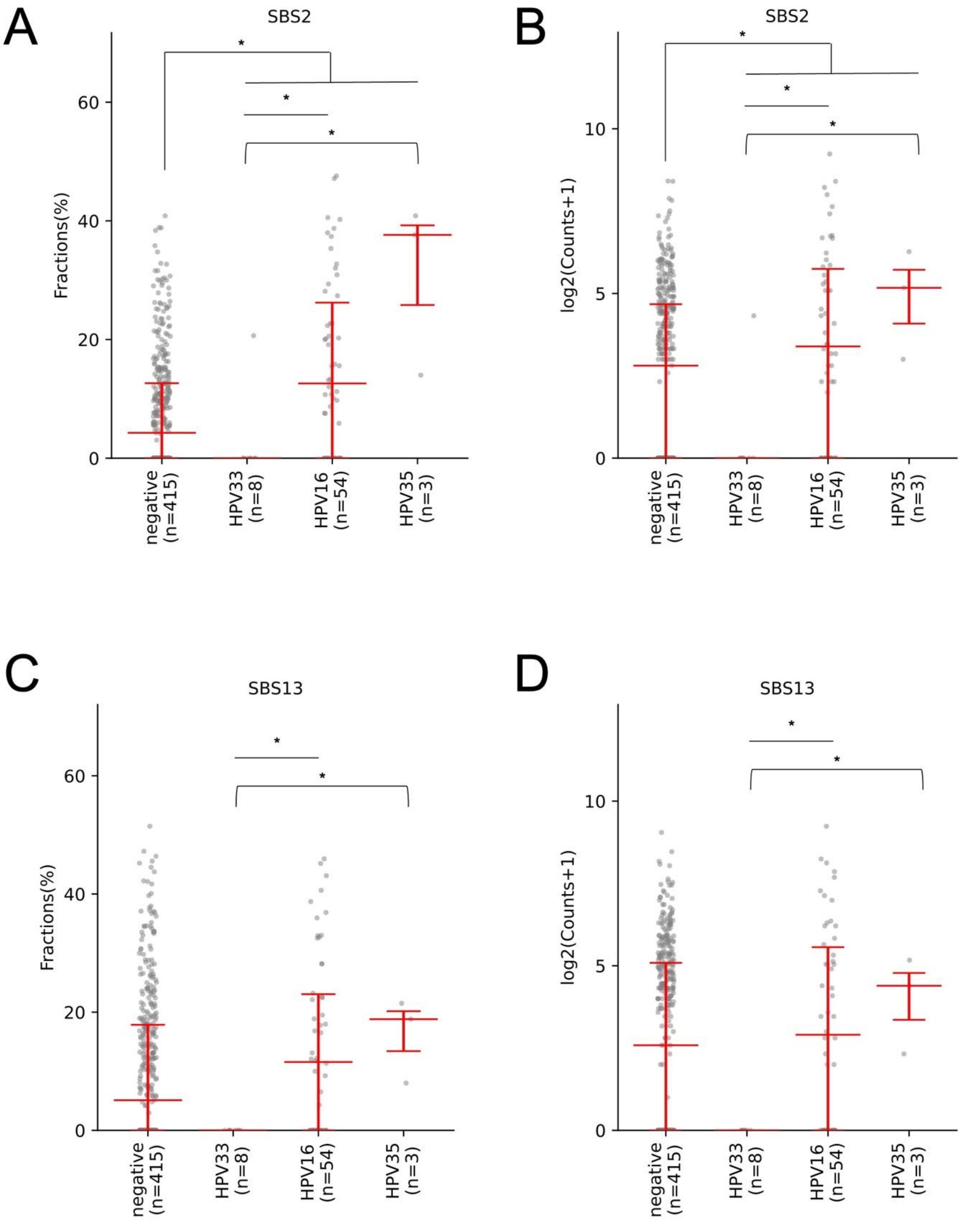

Fig. S7. HNSCC HPV strain differences in A,C) fractions (%) and B,D) log2(mutation counts+1) of SBS2 and SBS13 (both APOBEC associated signatures). Data are presented as median values with interquartile range (25th–75th percentile). * two-sided MWU test, $p < 0.05$. Source data are provided as a Source Data file.

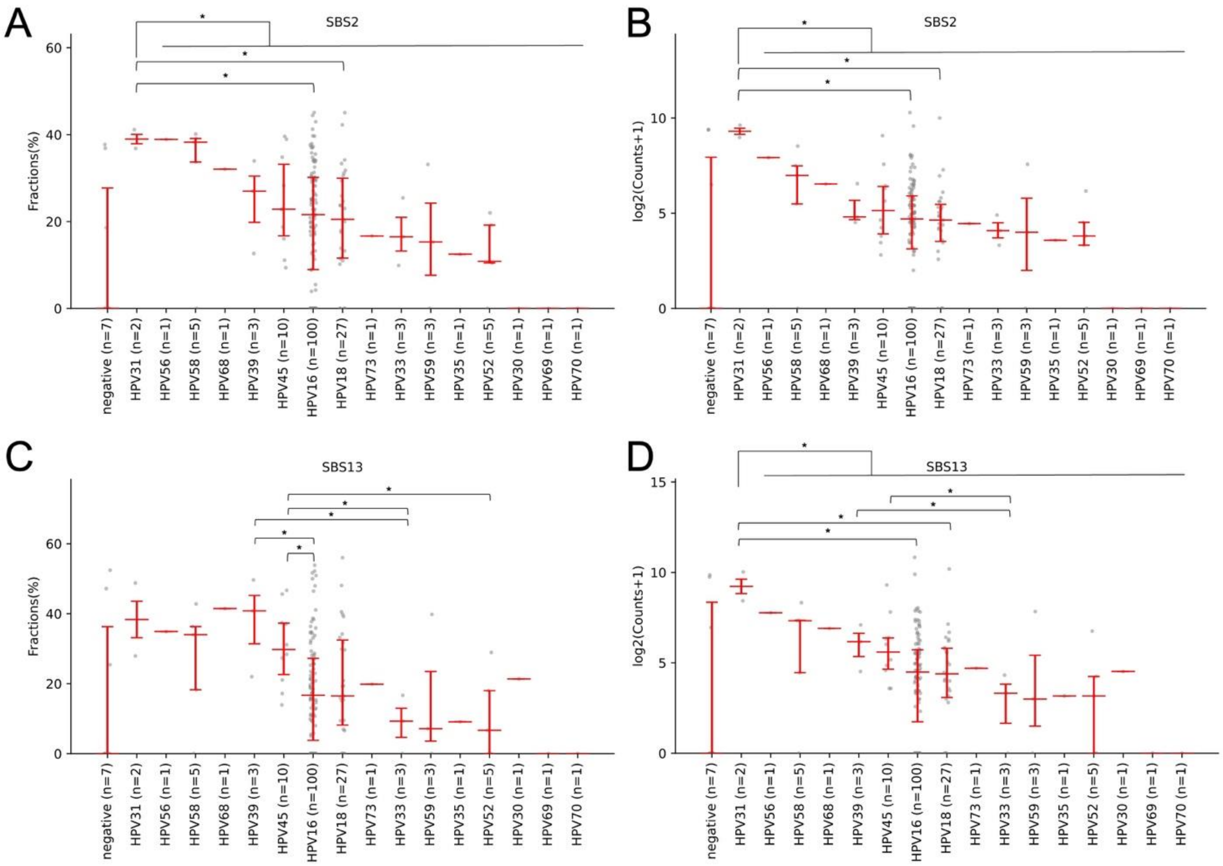

Fig. S8. Cervical cancer HPV strain differences in A,C) fractions (%) and B,D) log2(mutation counts+1) of SBS2 and SBS13 (both APOBEC associated signatures). Data are presented as median values with interquartile range (25th–75th percentile). * two-sided MWU test, $p < 0.05$. Source data are provided as a Source Data file.

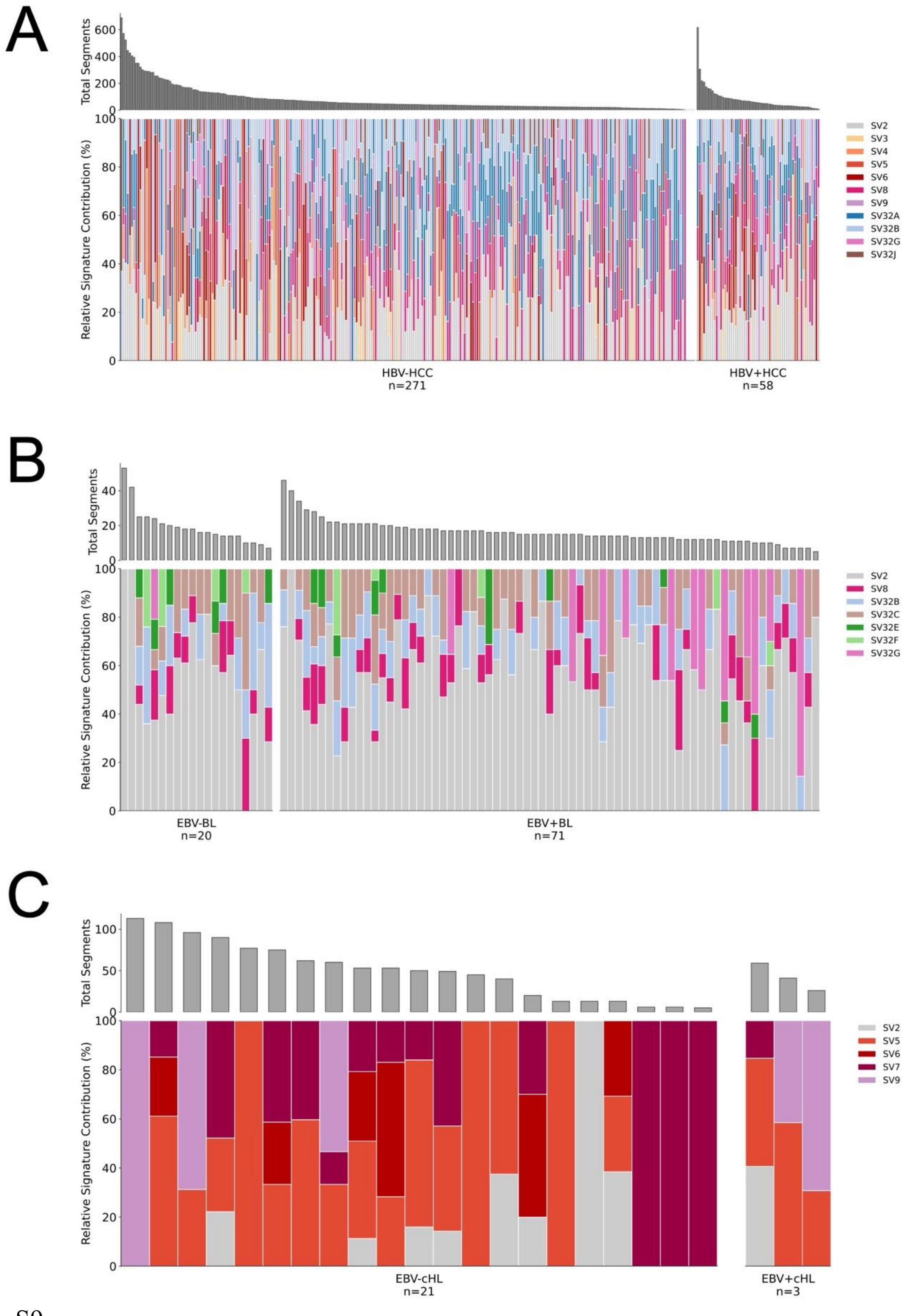

A
Total Segments
Relative Signature Contribution (%)
HBV-HCC
n=271
HBV+HCC
n=58
SV2
SV3
SV4
SV5
SV6
SV8
SV9
SV32A
SV32B
SV32G
SV32J
B
Total Segments
Relative Signature Contribution (%)
EBV-BL
n=20
EBV+BL
n=71
SV2
SV8
SV32B
SV32C
SV32E
SV32F
SV32G
C
Total Segments
Relative Signature Contribution (%)
EBV-cHL
n=21
EBV+cHL
n=3
SV2
SV5
SV6
SV7
SV9

Fig. S9.

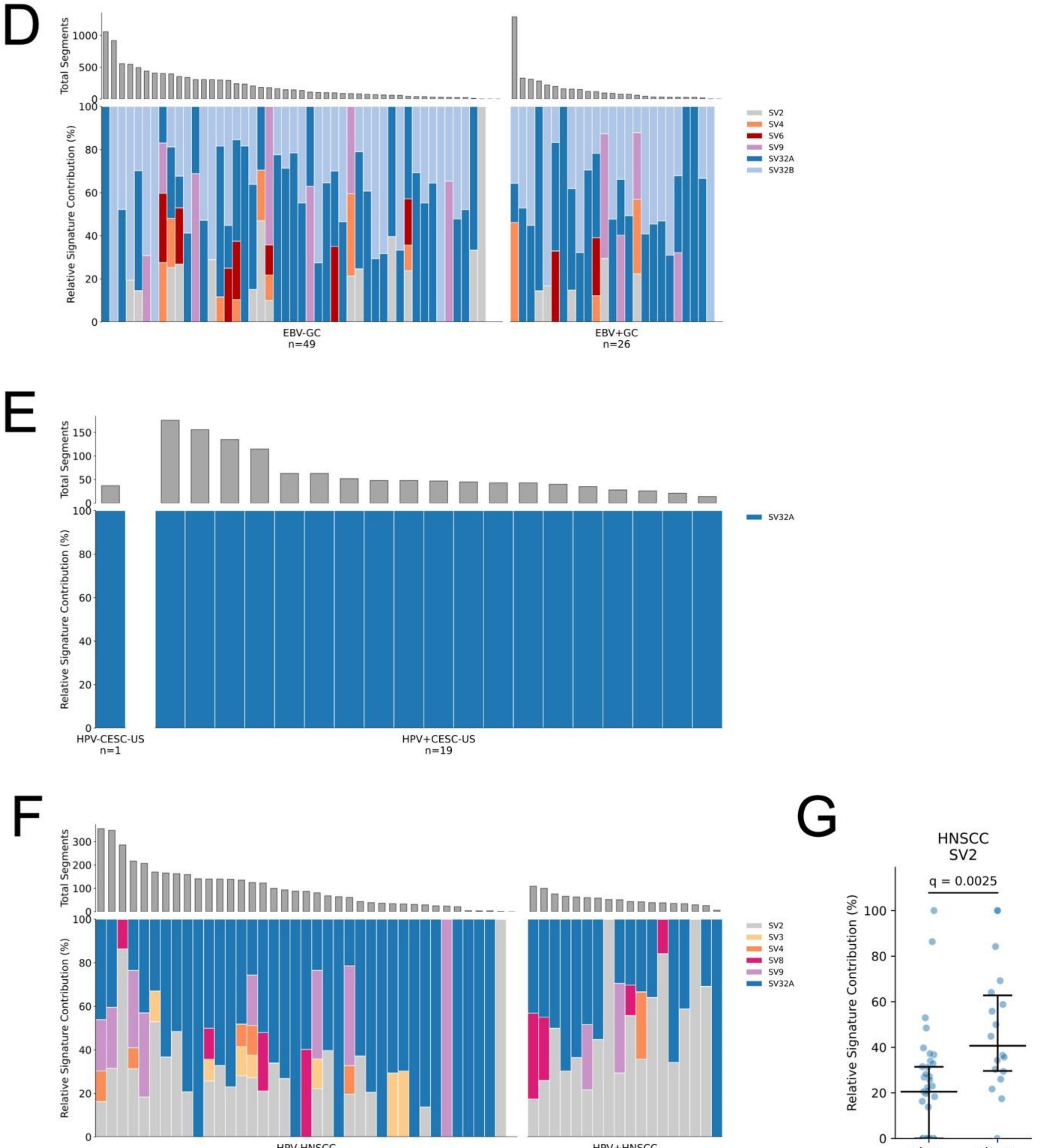

Fig. S9 (continued). Structure variant signatures in A) HCC, B) BL, C) cHL, D) GC, E) CC, F) HNSCC. G) Significant structure variant signature in HNSCC. Q value by two-sided MWU test, BH corrected. Data are presented as median values with interquartile range (25th–75th percentile). GC, gastric cancer; HCC, hepatocellular carcinoma; cHL, classical hodgkin lymphoma; HNSCC, head and neck squamous cell carcinoma; CESC, cervical cancer; BL, Burkitt lymphoma.

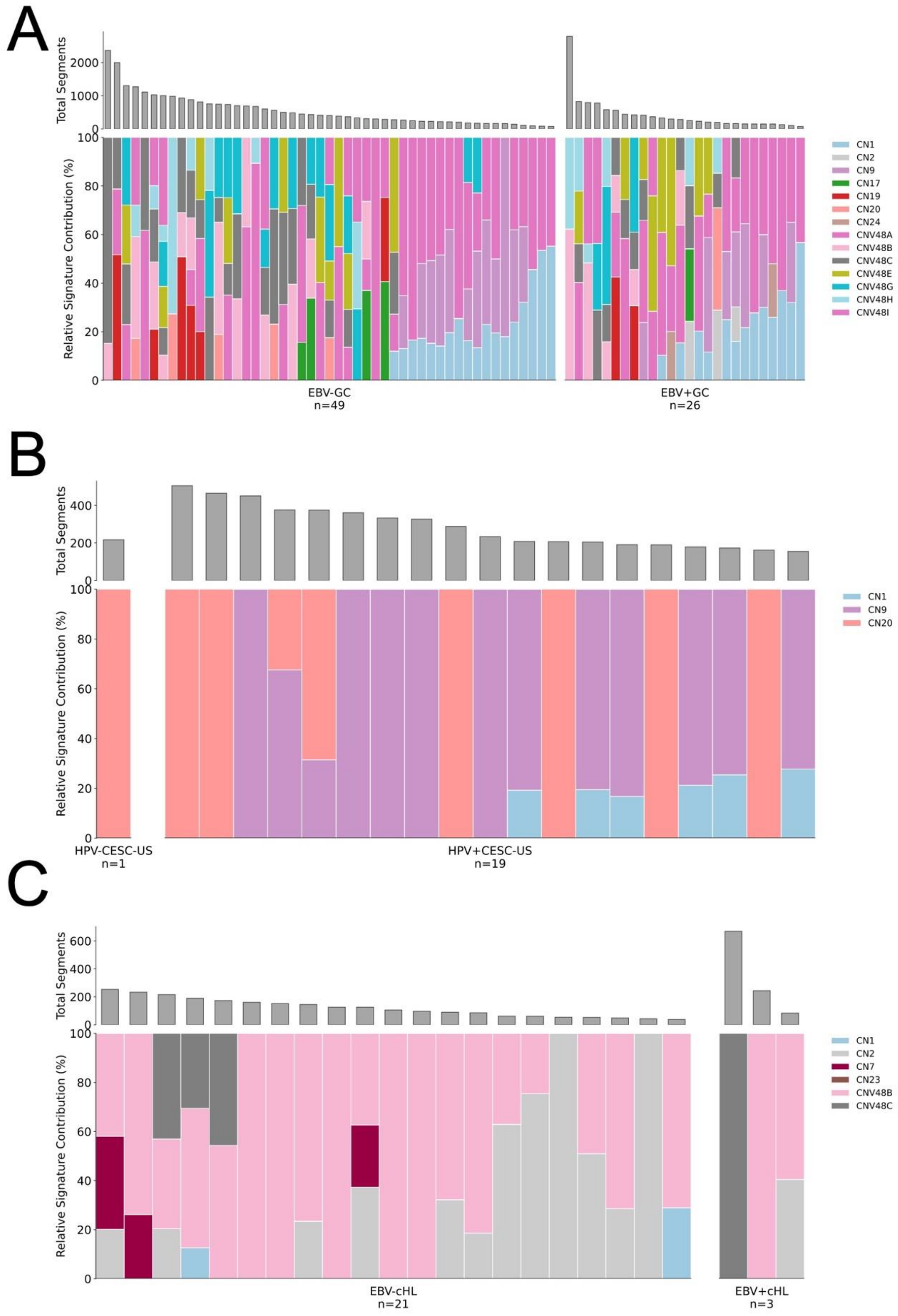

A
Total Segments
Relative Signature Contribution (%)
EBV-GC
n=49
EBV+GC
n=26
CN1
CN2
CN9
CN17
CN19
CN20
CN24
CNV48A
CNV48B
CNV48C
CNV48E
CNV48G
CNV48H
CNV48I
B
HPV-CESC-US
n=1
HPV+CESC-US
n=19
CN1
CN9
CN20
C
EBV-cHL
n=21
EBV+cHL
n=3
CN1
CN2
CN7
CN23
CNV48B
CNV48C

Fig. S10.

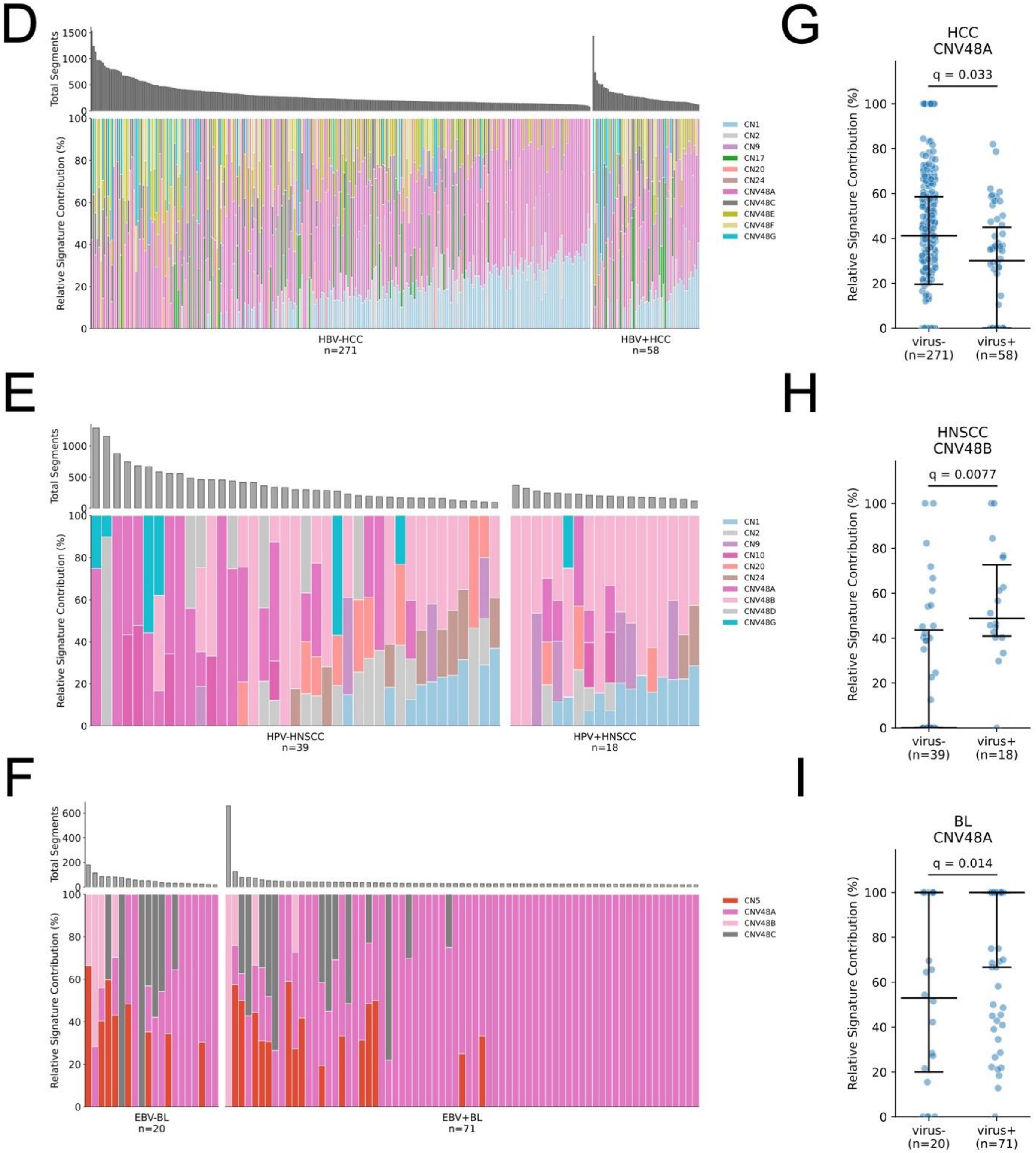

Fig. S10 (continued). Copy number signatures in A) GC, B) CC, C) cHL, D) HCC, E) HNSCC, F) BL. G-I) Significant copy number signatures in G) HCC, H) HNSCC, and I) BL. Q value by two-sided MWU test, BH corrected. Data are presented as median values with interquartile range (25th–75th percentile). GC, gastric cancer; HCC, hepatocellular carcinoma; cHL, classical hodgkin lymphoma; HNSCC, head and neck squamous cell carcinoma; CESC, cervical cancer; BL, Burkitt lymphoma.

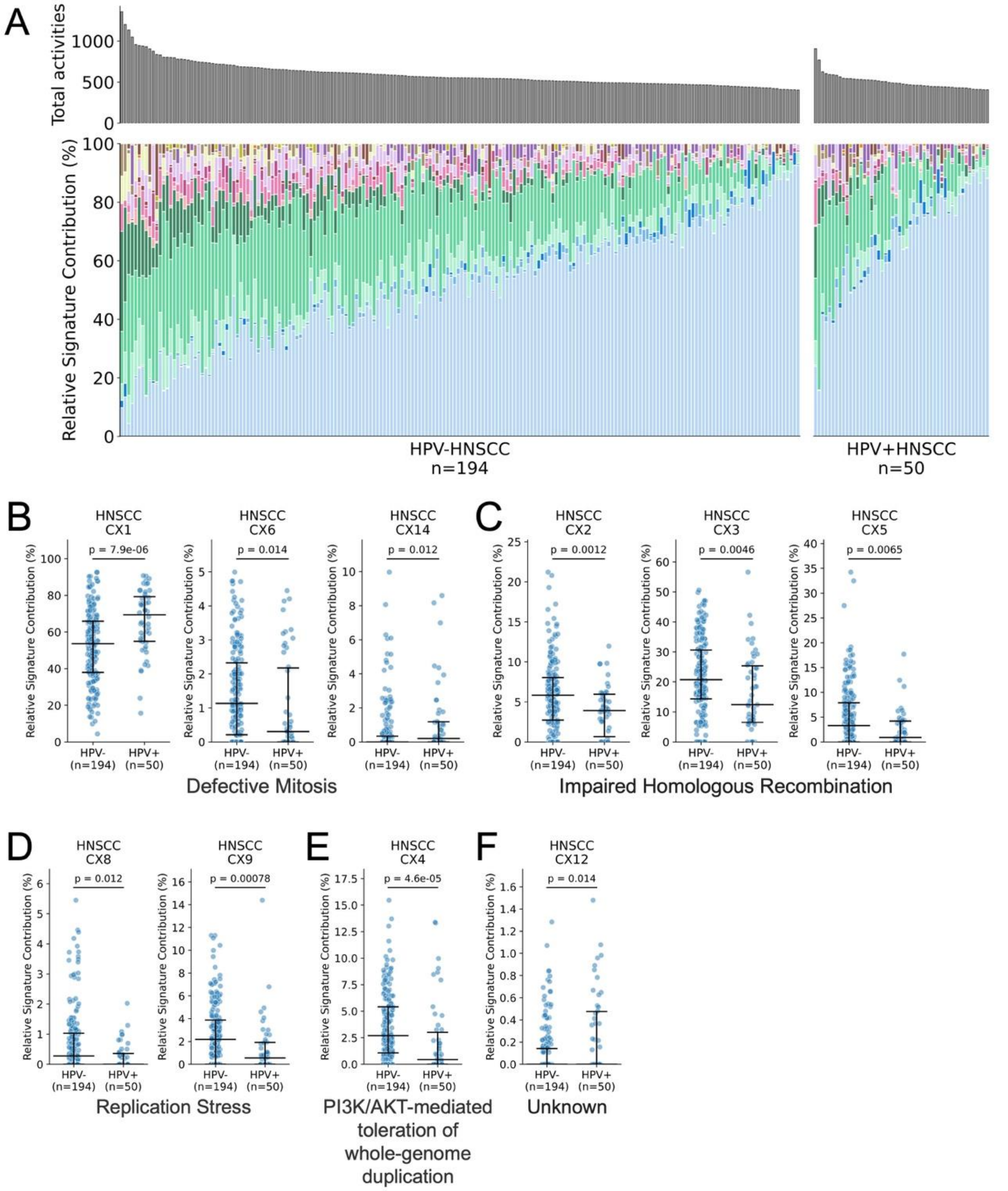

Fig. S11. Chromosomal instability signatures in A) HNSCC. B-F) Significant chromosomal instability signatures in HNSCC. P value by two-sided MWU test. Data are presented as median values with interquartile range (25th–75th percentile). Source data are provided as a Source Data file.

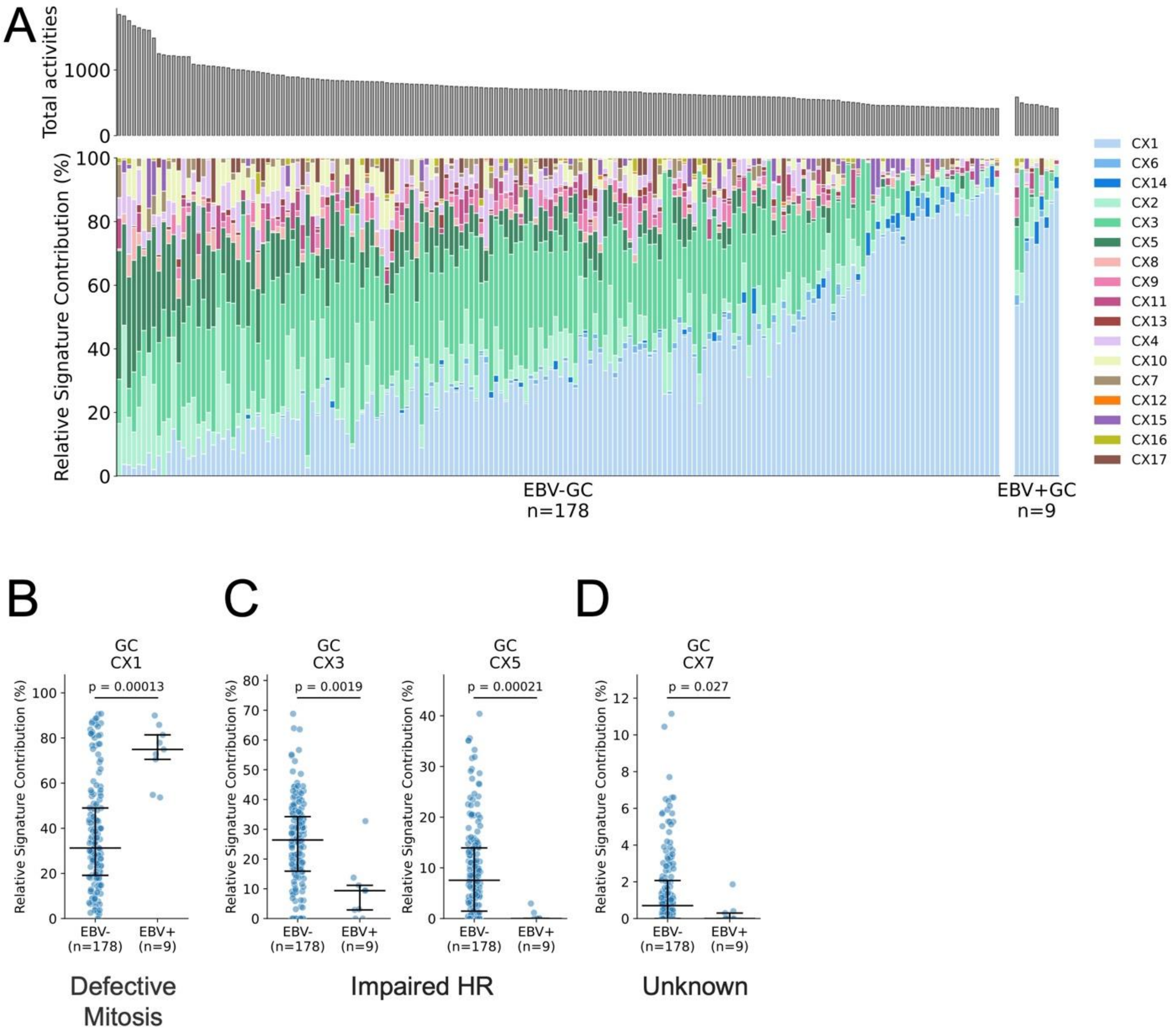

A
Total activities
1000
0
Relative Signature Contribution (%)
100
80
60
40
20
0
CX1
CX6
CX14
CX2
CX3
CX5
CX8
CX9
CX11
CX13
CX4
CX10
CX7
CX12
CX15
CX16
CX17
EBV-GC
n=178
EBV+GC
n=9
B
GC
CX1
p = 0.00013
EBV-
(n=178)
EBV+
(n=9)
Defective Mitosis
C
GC
CX3
p = 0.0019
GC
CX5
p = 0.00021
Impaired HR
D
GC
CX7
p = 0.027
Unknown

Fig. S12.

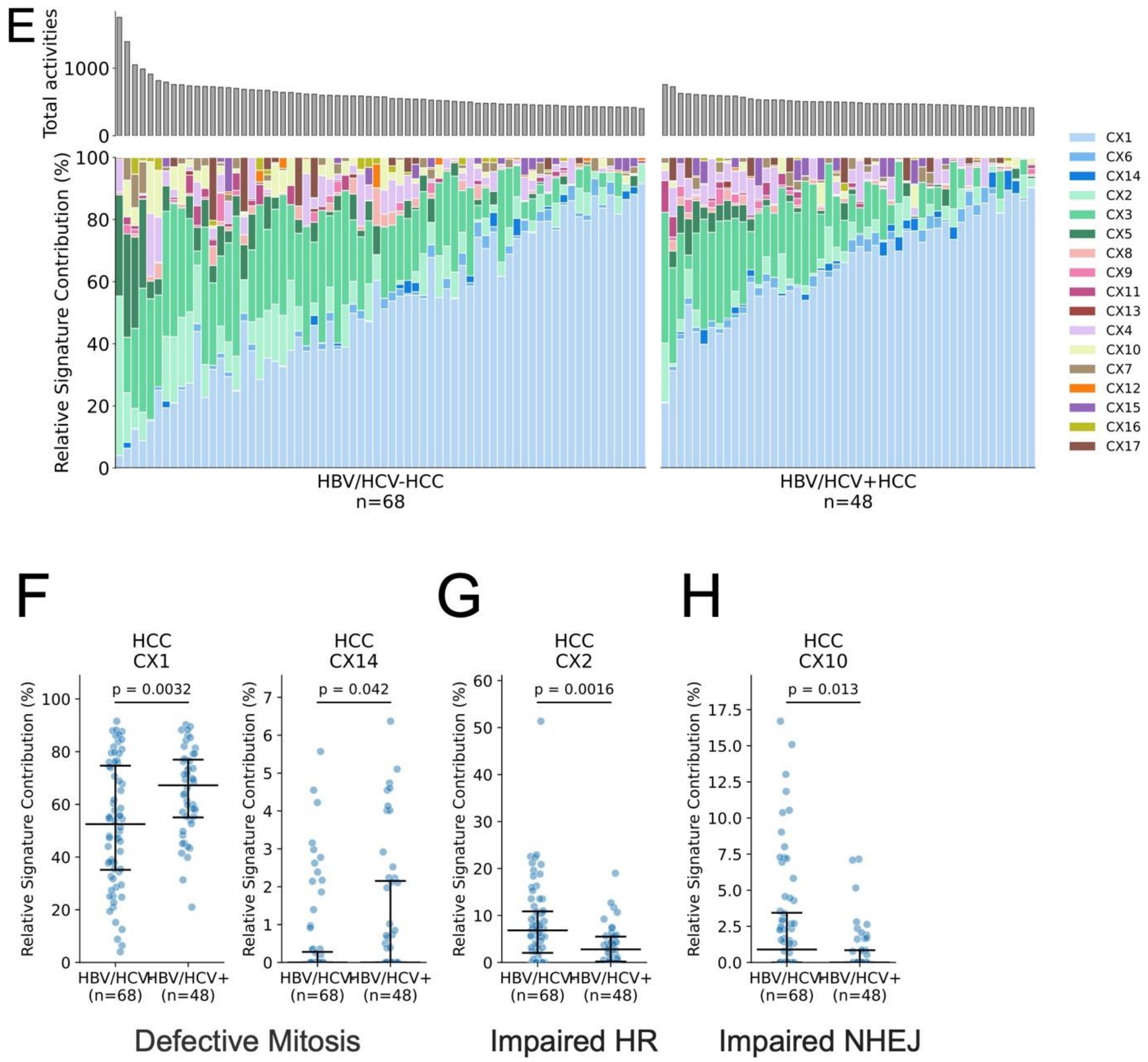

Fig. S12 (continued).

I

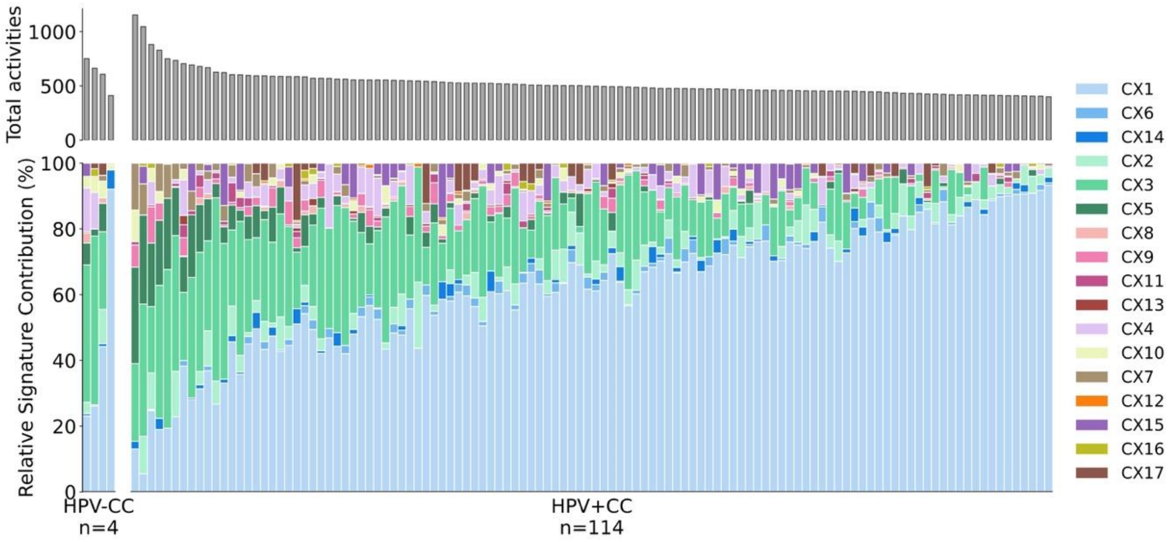

J

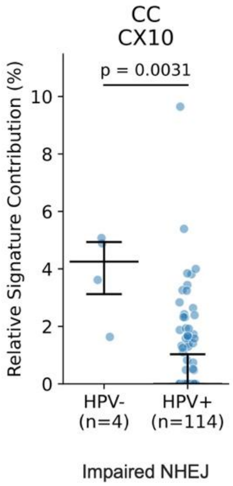

Fig. S12 (continued). Chromosomal instability signatures in A) GC, E) HCC, I) CC. B-D) Significant chromosomal instability signatures in GC. F-H) Significant chromosomal instability signatures in HCC. J) Significant chromosomal instability signatures in CC. P value by two-sided MWU test. Data are presented as median values with interquartile range (25th–75th percentile). Source data are provided as a Source Data file. GC, gastric cancer; HCC, hepatocellular carcinoma; CC, cervical cancer.

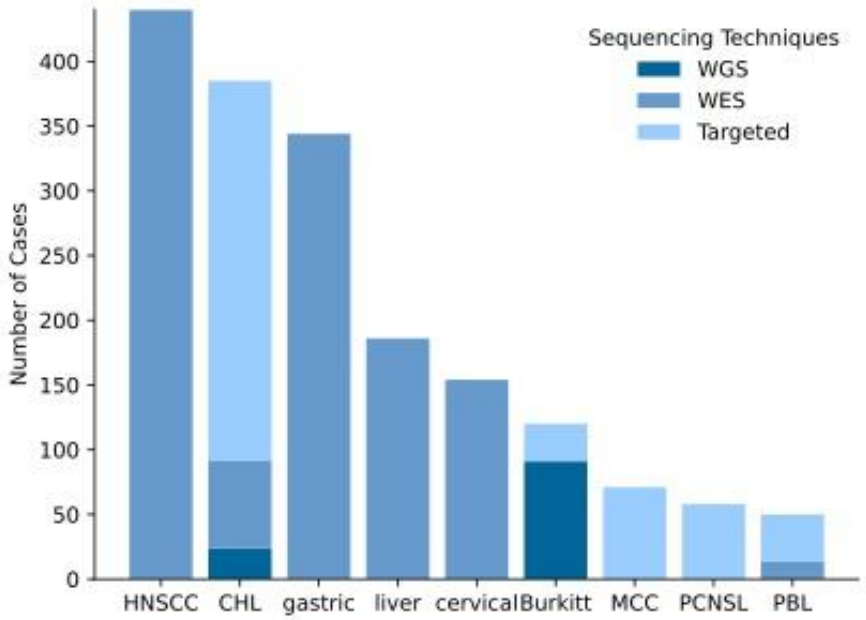

Fig. S13. Number of cases by each cancer types used in virus-associated-mutated-gene analysis colored by sequencing technologies. cHL, classical hodgkin lymphoma; HNSCC, head and neck squamous cell carcinoma; MCC, Merkel cell carcinoma; PBL, plasmablastic lymphoma; PCNSL, primary central nervous system lymphoma.

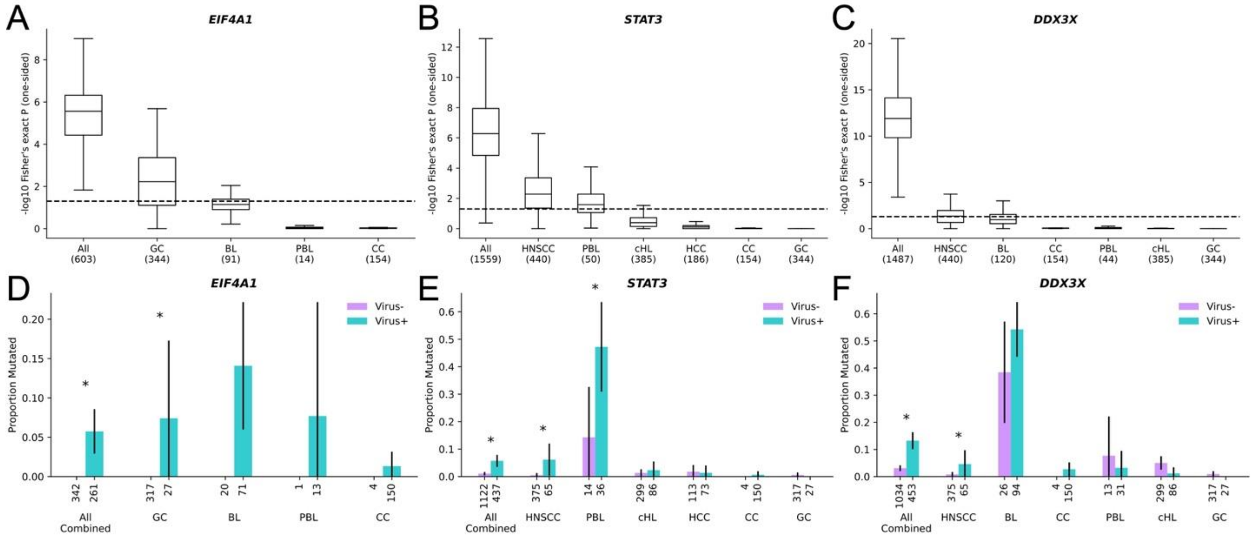

Fig. S14. Combined and individual cancer-type trends in virus-positive-associated genes. A–C) Boxplots of bootstrapped (n = 10,000) one-sided Fisher's exact p-values (direction: higher mutation rates in virus-positive cases) for each cancer type included in the combined analysis of each gene. Number of samples used in the analysis for each cancer type are shown in parenthesis. D–F) Differences in mutation proportions between virus-positive and virus-negative cases for each cancer type. GC, gastric cancer; HCC, hepatocellular carcinoma; cHL, classical hodgkin lymphoma; HNSCC, head and neck squamous cell carcinoma; MCC, Merkel cell carcinoma; PBL, plasmablastic lymphoma; CC, cervical cancer; BL, Burkitt lymphoma. * two-sided MWU test, $p < 0.05$. Data are presented as median values with interquartile range (25th–75th percentile). Source data are provided as a Source Data file.

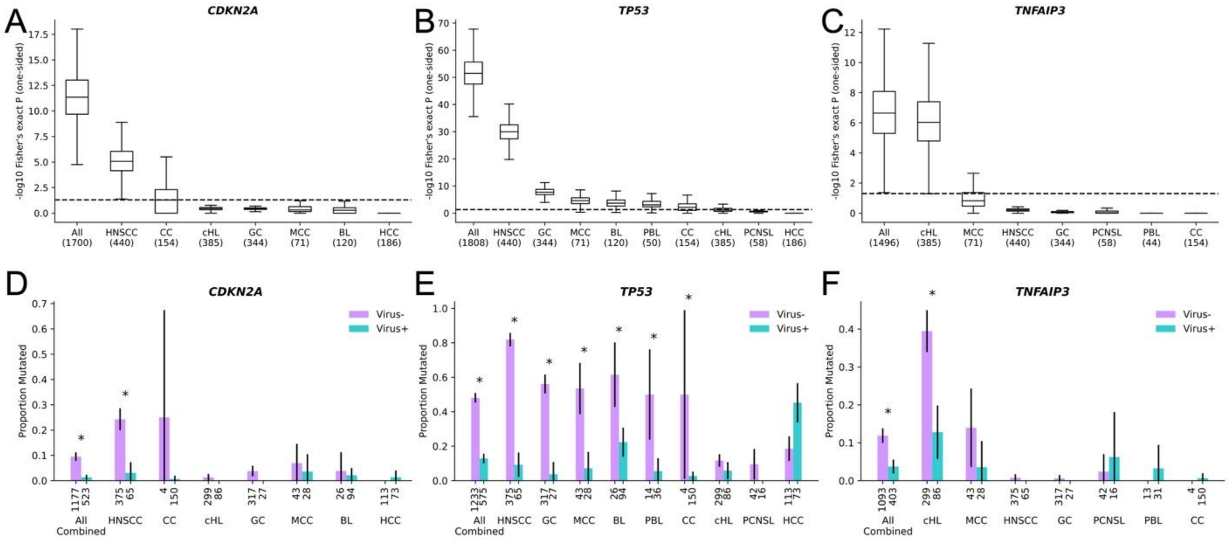

Fig. S15. Combined and individual cancer-type trends in virus-negative-associated genes. A–C) Boxplots of bootstrapped (n = 10,000) one-sided Fisher's exact p-values (direction: higher mutation rates in virus-negative cases) for each cancer type included in the combined analysis of each gene. Number of samples used in the analysis for each cancer type are shown in parenthesis. D–F) Differences in mutation proportions between virus-positive and virus-negative cases for each cancer type. GC, gastric cancer; HCC, hepatocellular carcinoma; cHL, classical hodgkin lymphoma; HNSCC, head and neck squamous cell carcinoma; MCC, Merkel cell carcinoma; PBL, plasmablastic lymphoma; PCNSL, primary central nervous system lymphoma; CC, cervical cancer; BL, Burkitt lymphoma. * two-sided MWU test, $p < 0.05$. Data are presented as median values with interquartile range (25th–75th percentile). Source data are provided as a Source Data file.

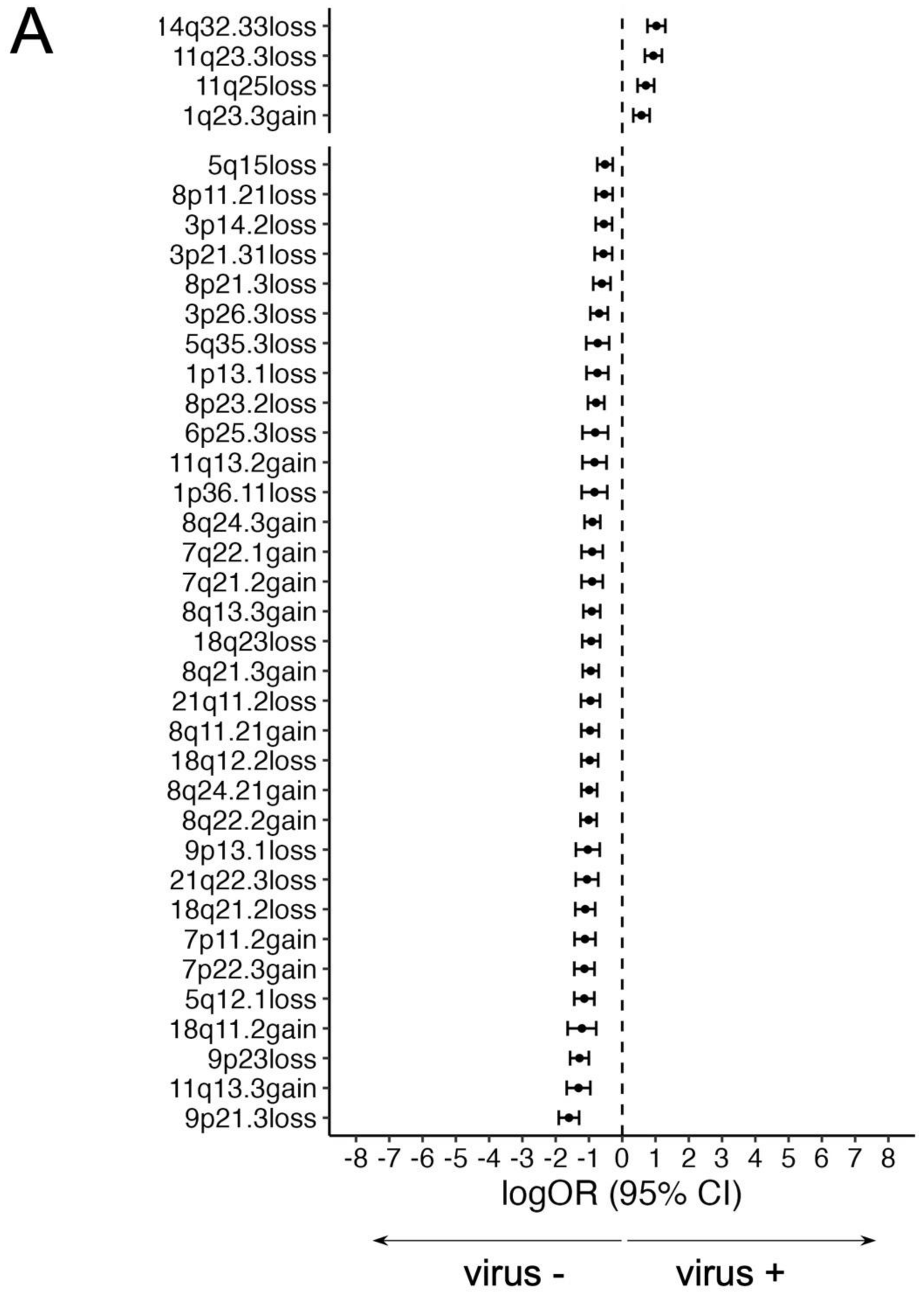
A
14q32.33loss
11q23.3loss
11q25loss
1q23.3gain
5q15loss
8p11.21loss
3p14.2loss
3p21.31loss
8p21.3loss
3p26.3loss
5q35.3loss
1p13.1loss
8p23.2loss
6p25.3loss
11q13.2gain
1p36.11loss
8q24.3gain
7q22.1gain
7q21.2gain
8q13.3gain
18q23loss
8q21.3gain
21q11.2loss
8q11.21gain
18q12.2loss
8q24.21gain
8q22.2gain
9p13.1loss
21q22.3loss
18q21.2loss
7p11.2gain
7p22.3gain
5q12.1loss
18q11.2gain
9p23loss
11q13.3gain
9p21.3loss
-8 -7 -6 -5 -4 -3 -2 -1 0 1 2 3 4 5 6 7 8
logOR (95% CI)
virus -
virus +

Fig. S16.

B

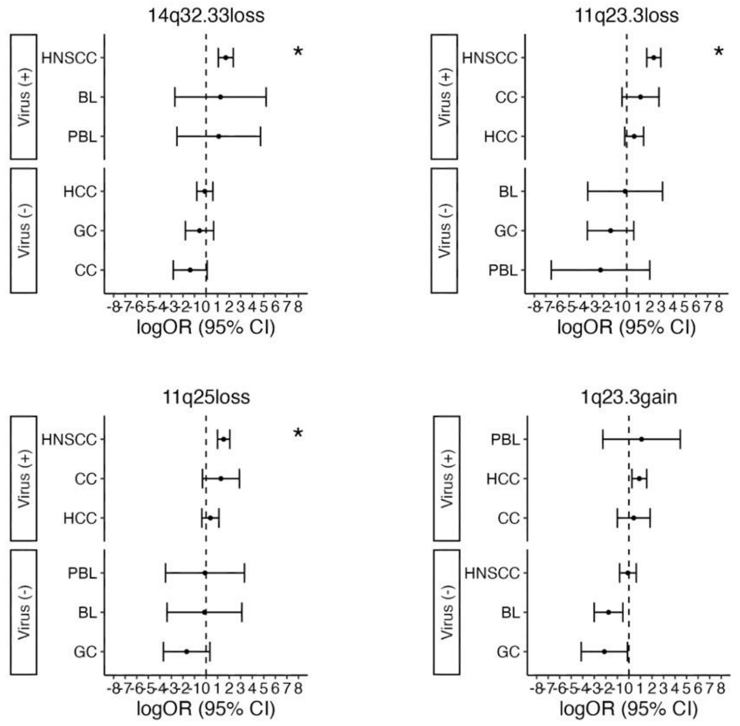

C

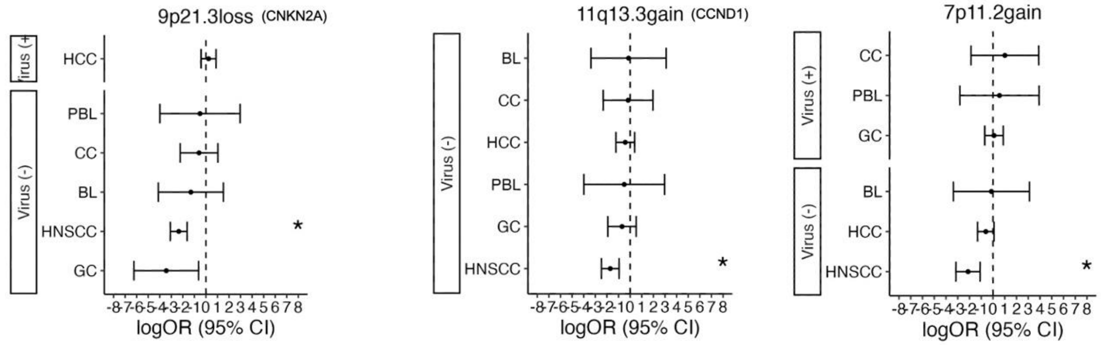

Fig. S16 (continued). A) Odds ratio of CNA in virus positive versus virus negative tumors in the combined cohort. B-C) Odds ratio of CNA in virus positive versus virus negative tumors by cancer type in regions with a significant OR of CNA in B) virus positive tumors and C) virus negative tumors from the combined cohort and the top ranking region with a significant OR of CNA in virus negative tumors from the combined cohort. Data are presented as log(odds ratio) values with error bars indicating 95% confidence intervals. GC, gastric cancer; HCC, hepatocellular carcinoma; cHL, classical hodgkin lymphoma; HNSCC, head and neck squamous cell carcinoma; MCC, Merkel cell carcinoma; PBL, plasmablastic lymphoma; CC, cervical cancer; BL, Burkitt lymphoma. * $q < 0.0001$, two-sided chi-square test, BH corrected.

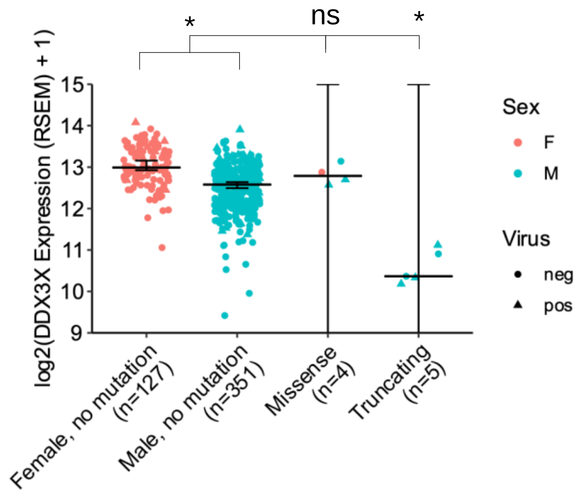

Fig. S17. Expression of *DDX3X* and *DDX3X* mutation status in TCGA-HNSC (n = 487). * two-sided MWU test, $p < 0.05$. Data are presented as median values with interquartile range (25th–75th percentile). ns, not significant. RSEM,

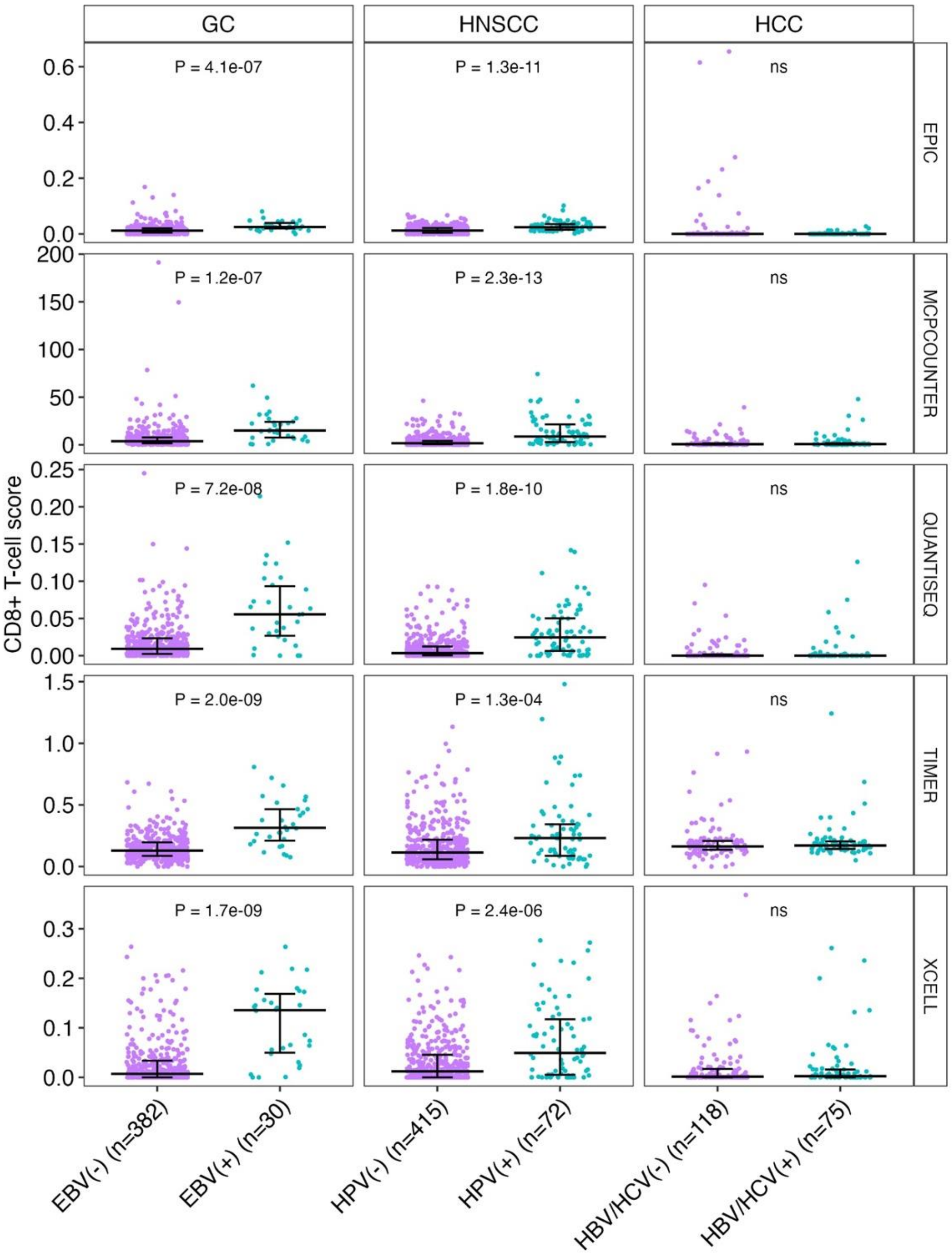

Fig. S18. CD8+ T cell infiltration in virus positive and negative GC, HNSCC and HCC TCGA tumors, across multiple deconvolution algorithms. GC, gastric cancer; HCC, hepatocellular carcinoma; HNSCC, head and neck squamous cell carcinoma. P values from two-sided MWU test. Data are presented as median values with interquartile range (25th–75th percentile). Source data are provided as a Source Data file.

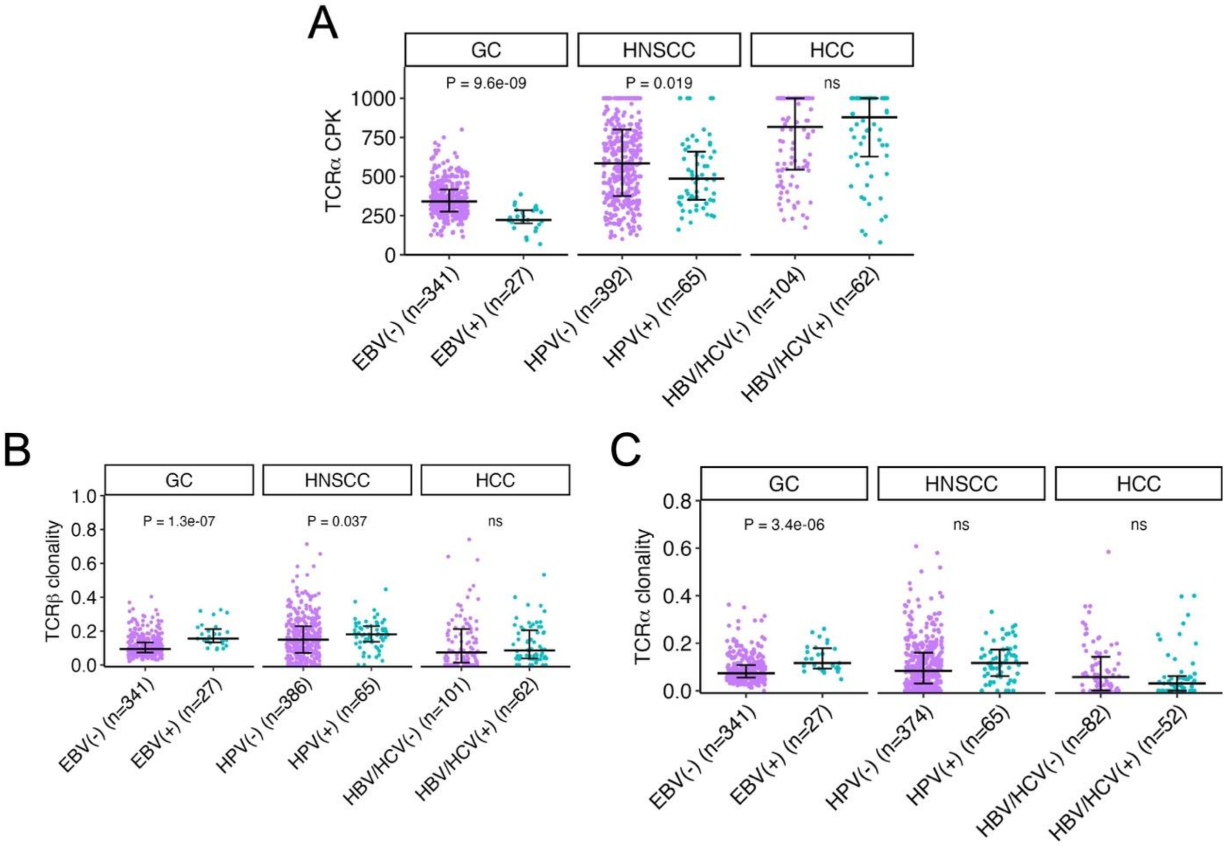

Fig. S19. Analysis of TCR alpha and beta CDR3 diversity metrics in virus positive and negative GC, HNSCC and HCC TCGA tumors. A) TCRα clonotypes per thousand reads (CPK). B) TCRβ and C) TCRα clonality, defined as 1 - normalized Shannon entropy. GC, gastric cancer; HCC, hepatocellular carcinoma; HNSCC, head and neck squamous cell carcinoma. Data are presented as median values with interquartile range (25th–75th percentile). $p < 0.05$, two-sided MWU test. Source data are provided as a Source Data file.
