# Supplementary material for: Genomic landscape of virus-associated cancers
Source: Nat Commun. 2025 Jul 1;16:5887. doi: 10.1038/s41467-025-60836-9 (PMC12219571; doi:10.1038/s41467-025-60836-9)
Supplement: Supplementary file 3 — Description of Additional Supplementary Files [file 41467_2025_60836_MOESM3_ESM.pdf]

<u>Description of Additional Supplementary Files</u>

Supplementary Data 1. Studies included in Fig. 1A (M/F incidence rates).

Supplementary Data 2. Studies included in Fig. 1B (M/F incidence rates on virus positive vs negative).

Supplementary Data 3. Clinical characteristics of patients in study.

Supplementary Data 4. Driver genes used in each cancer types.

Supplementary Data 5. Median values of nonsynonymous mutations in virus-positive and virus-negative tumors in 9 cancers.

Supplementary Data 6. Association of sex, age, virus strains, cancer subtype on mutation load.

Supplementary Data 7. Mutational signature comparison on virus-positive and virus-negative cases.

Supplementary Data 8. Association of sex, age, virus strains, cancer subtype on mutational signatures.

Supplementary Data 9. Multivariable analysis (Negative Binomial Generalized Linear Model) on SBS2/APOBEC signature in HPV-positive HNSCC.

Supplementary Data 10. Multivariable analysis (Negative Binomial Generalized Linear Model) on APOBEC signatures (SBS2/13) in Cervical Cancer.

Supplementary Data 11. WGS cases used in structure variation and copy number signature analysis.

Supplementary Data 12. CIN signature comparison on virus-positive and virus-negative cases.

Supplementary Data 13. Association of sex, age, virus strains, cancer subtype on CIN signatures.

Supplementary Data 14. Recurrently mutated genes in virus-associated cancers compared to non-virus associated cancers (and vice versa).

Supplementary Data 15. Extended Burkitt lymphoma cohort for DDX3X and EBV association analysis.

Supplementary Data 16. Association of sex, age, virus strains, cancer subtype on 6 virus-status-associated genes.

Supplementary Data 17. Mutations in DDX3X and EIF4A1 in 2,488 tumors.

Supplementary Data 18. Studies included in Figure 5A (biomarkers of immunotherapy response).

Supplementary Data 19. Odds ratios shown in Figure 5A (biomarkers of immunotherapy response).

Supplementary Data 20. GISTIC peaks in virus-associated cancers.
