# Supplementary material for: Genomic landscape of virus-associated cancers
Source: Nat Commun. 2025 Jul 1;16:5887. doi: 10.1038/s41467-025-60836-9 (PMC12219571; doi:10.1038/s41467-025-60836-9)
Supplement: Supplementary file 5 — Reporting Summary [file 41467_2025_60836_MOESM5_ESM.pdf]

nature portfolio

Corresponding author(s): Raul Rabadan

Last updated by author(s): Apr 11, 2025

Reporting Summary

Nature Portfolio wishes to improve the reproducibility of the work that we publish. This form provides structure for consistency and transparency in reporting. For further information on Nature Portfolio policies, see our Editorial Policies and the Editorial Policy Checklist.

Statistics

For all statistical analyses, confirm that the following items are present in the figure legend, table legend, main text, or Methods section.

n/a	Confirmed	
☐	☒	The exact sample size (*n*) for each experimental group/condition, given as a discrete number and unit of measurement
☐	☒	A statement on whether measurements were taken from distinct samples or whether the same sample was measured repeatedly
☐	☒	The statistical test(s) used AND whether they are one- or two-sided<br>*Only common tests should be described solely by name; describe more complex techniques in the Methods section.*
☐	☒	A description of all covariates tested
☐	☒	A description of any assumptions or corrections, such as tests of normality and adjustment for multiple comparisons
☐	☒	A full description of the statistical parameters including central tendency (e.g. means) or other basic estimates (e.g. regression coefficient) AND variation (e.g. standard deviation) or associated estimates of uncertainty (e.g. confidence intervals)
☐	☒	For null hypothesis testing, the test statistic (e.g. *F*, *t*, *r*) with confidence intervals, effect sizes, degrees of freedom and *P* value noted<br>*Give P values as exact values whenever suitable.*
☒	☐	For Bayesian analysis, information on the choice of priors and Markov chain Monte Carlo settings
☒	☐	For hierarchical and complex designs, identification of the appropriate level for tests and full reporting of outcomes
☐	☒	Estimates of effect sizes (e.g. Cohen's *d*, Pearson's *r*), indicating how they were calculated

Our web collection on statistics for biologists contains articles on many of the points above.

Software and code

Policy information about availability of computer code

Data collection	Public data was downloaded from corresponding websites or publications, which are detailed in the Methods section.
Data analysis	All analyses were performed using publicly available software: for alignment of Kaposi sarcoma sample WES data to GRCh37, Burrows-Wheeler aligner v.0.7.17 (https://github.com/lh3/bwa); for calling somatic variants, SAVI version 2 (https://github.com/WinterLi1993/SAVI); for calling mutation signatures from somatic variants, SigProfilerExtractor v.1.1.24 (https://github.com/AlexandrovLab/SigProfilerExtractor); for copy number segmentation of Kaposi sarcoma samples, Sequenza (https://sequenzatools.bitbucket.io/); for defining significant regions of recurrent CNAs, GISTIC version 2.0 (https://broadinstitute.github.io/gistic2/); for combining p-values using the weighted Fisher method (wFisher), metapro (http://github.com/unistbig/metapro). For general coding, R (version 4.4.1) and Python (version 3.9.21) were used. Detailed information on used software is also provided in the respective sections of the Methods.

For manuscripts utilizing custom algorithms or software that are central to the research but not yet described in published literature, software must be made available to editors and reviewers. We strongly encourage code deposition in a community repository (e.g. GitHub). See the Nature Portfolio guidelines for submitting code & software for further information.

Data

Policy information about availability of data

All manuscripts must include a data availability statement. This statement should provide the following information, where applicable:

- Accession codes, unique identifiers, or web links for publicly available datasets
- A description of any restrictions on data availability
- For clinical datasets or third party data, please ensure that the statement adheres to our policy

WES raw data (FASTQ and BAM files) of Kaposi sarcoma generated in this study have been deposited in the European Nucleotide Archive (ENA) at EMBL-EBI database under accession code PRJEB76508 [https://www.ebi.ac.uk/ena/browser/view/PRJEB76508]. The TCGA (HNSCC, CC and GC) cohort's clinical data, mutation and copy number alteration calls are available at cBioportal (HNSCC:https://www.cbioportal.org/study/summary?id=hnsc_tcga, CC:https://www.cbioportal.org/study/summary?id=cesc_tcga_pan_can_atlas_2018, GC:https://www.cbioportal.org/study/summary?id=stad_tcga), raw and normalized chromosomal instability (CIN) signature activities are available within the supplementary information of Drews et al.'s publication139. SV and CN data of PCAWG WGS cases are available from The International Cancer Genome Consortium Accelerating Research in Genomic Oncology (ICGC ARGO) data platform (Legacy ICGC 25K Data, https://docs.icgc-argo.org/docs/data-access/icgc-25k-data#open-release-data---object-bucket-details; SV: s3://icgc25k-open/PCAWG/consensus_sv/; CN: s3://icgc25k-open/PCAWG/consensus_cnv/). Clinical and genomic data including SNV, CN, and/or SV calls of other cancer types are available within the supplementary information of their published studies21,105,114-117,119-123,152-155,188,189. EBV status of the Hodgkin lymphoma cohort from Alig et al.123 has been acquired from the authors directly. Source data are provided with this paper.

Research involving human participants, their data, or biological material

Policy information about studies with human participants or human data. See also policy information about sex, gender (identity/presentation), and sexual orientation and race, ethnicity and racism.

Reporting on sex and gender	Our study primarily utilized publicly available datasets where sex information was already provided. No sex and/or gender was considered in the study design. For 10 Kaposi cases, sex was based on self-report.
Reporting on race, ethnicity, or other socially relevant groupings	Our study do not report on race, ethnicity, or other socially relevant groupings
Population characteristics	Our study utilized publicly available datasets, which included demographic and clinical characteristics such as age, sex, and cancer type. No additional patient data were collected or processed beyond what was provided in the original datasets. In addition to publicly available datasets, we included 10 Kaposi sarcoma samples, which were sequenced when samples were available.
Recruitment	For the 10 Kaposi sarcoma samples, sequencing was performed on available samples without specific recruitment criteria. No additional participant selection was conducted, and sample availability determined inclusion.
Ethics oversight	All biological samples from Kaposi patients (tissue and blood specimens) were obtained after the written consent from the patients and the sample IDs are anonymized. The study was approved by the Committee for the Ethics of the Research and Bioethics of the National Research Council (CNR n.12629)

Note that full information on the approval of the study protocol must also be provided in the manuscript.

Field-specific reporting

Please select the one below that is the best fit for your research. If you are not sure, read the appropriate sections before making your selection.

☒ Life sciences ☐ Behavioural & social sciences ☐ Ecological, evolutionary & environmental sciences

For a reference copy of the document with all sections, see nature.com/documents/nr-reporting-summary-flat.pdf

Life sciences study design

All studies must disclose on these points even when the disclosure is negative.

Sample size	No sample size calculation was performed since all available samples were used.
Data exclusions	To eliminate noise from hypermutated samples, for all cancers, only cases with <300 mutations were included for analysis of recurrently mutated genes in virus-associated cancers
Replication	There was no experimental findings in this study.
Randomization	Most of the samples involved in this study (except the new 10 Kaposi samples) are from previous publications. Studies were selected if genomic data was publicly available. Sex or age was not a criterion for study selection.

Blinding

In our study, the analysis is retrospective and relies on pre-existing genomic data from tumors, eliminating the risk of allocation bias. Thus, blinding was not necessary for this research.

Reporting for specific materials, systems and methods

We require information from authors about some types of materials, experimental systems and methods used in many studies. Here, indicate whether each material, system or method listed is relevant to your study. If you are not sure if a list item applies to your research, read the appropriate section before selecting a response.

Materials & experimental systems

n/a	Involved in the study
☒	☐ Antibodies
☒	☐ Eukaryotic cell lines
☒	☐ Palaeontology and archaeology
☒	☐ Animals and other organisms
☒	☐ Clinical data
☒	☐ Dual use research of concern
☒	☐ Plants

Methods

n/a	Involved in the study
☒	☐ ChIP-seq
☒	☐ Flow cytometry
☒	☐ MRI-based neuroimaging

Plants

Seed stocks

No plants were utilized in our study

Novel plant genotypes

No novel plant genotypes were produced in our study

Authentication

No plant authentication were included in this study
